# Registration: A Word to Nurses

**Published:** 1888-03-17

**Authors:** 


### The Hospital

A Weekly Institutional Journal of

Science, Medicine, and Philanthropy.

*For Hospitals, Asylums, Medical Practitioners, Students, Nurses, Families, Parishes, Congregations, and the Charitable Public.*

Vol. III. No. 77.] [March 17, 1888.

### Registration: A Word to Nurses.

There is an uneasy feeling abroad in the hospital and nursing worlds that a crisis in the history of nurses is fast approaching. Many portentous signs are visible in the professional skies which fill weak and ignorant spirits with alarm, but which do not produce the same effect upon those who look behind the scenes, and who know the exact value of resolutions passed by voluntary associations and endowed with the fleeting immortality of printer's ink. It is well to remember that Governments in civilised countries are always slow to interfere with vested interests, even when those interests are of a doubtfully legitimate character. No one can say that the right of a practising nurse, however trained, to continue to earn her living by her own calling until the end of her life, is other than a perfectly legitimate vested interest. That right no reasonable association would venture to dispute; and any organisation which should presume to dispute it would obtain no help from any Government, whatever its *personnel* or its politics might be.

This important certainty might be made to appear much more evident by innumerable historical examples. One such will suffice. At the beginning of the present century the medical profession was in a condition precisely similar to that in which nurses now find themselves. Many physicians and surgeons possessed the highest academical qualifications then obtainable. Many had qualifications of a less unimpeachable, but still of a positive value. A large number had no qualification at all, except this, that they were in actual practice as medical men, and depended for their livelihood upon such practice. In the year 1815 a Medical Register was formed, similar to that which is now proposed for nurses. On that Register was placed not only every man of the highest and every man of the lowest qualification, but every man of no academical qualification of any kind, who could prove that he was in the actual practice of medicine at the date of the opening of the Register. This example, in its manifest justice, may soothe the alarms of any and of all who are trembling for their means of livelihood.

Matrons and nurses being thus in a position of unquestionable security, may put away their doubts and fears, and assume such an attitude of mind as the impending crisis demands. That attitude should be one of critical watchfulness and broad intelligence. Every matron and nurse must bear in mind that any rules and regulations which may obtain the authority of law will be rules and regulations which affect *her* individually; that any powers which may be conferred upon a registering organisation will be powers which may be exercised upon *her;* powers which will define her position; which may and will in the last resort be powers of professional life and death. All intelligent nurses and matrons of course see this as clearly as we do; but it is essential that every nurse should see it, whether intelligent or not, and that everyone should be prepared to understand all rules and regulations proposed for her future government, and to support or to oppose them according as they may seem to her right or wrong, wise or unwise.

There are at the present moment two associations, working separately, each of which is desirous of promoting the registration of nurses. The older and more representative of the two is *The Hospitals Association*, which, a few years ago, began to organise the matrons and nurses. From it has sprung the younger, the *British Nurses Association,* which has come into existence during the last few months. The Hospitals Association was established five years ago on the wider—indeed, on the widest possible—basis, including as it does not only matrons, sisters, nurses, and doctors, but treasurers, secretaries, members of committees, subscribers, and hospital managers of every rank and degree. The British Nurses Association, the active promoters of which seceded from the parent association so recently as last November, seems to make a strong point of the fact, that the only classes to be represented in its management and organisation are to be members of the two professions of nursing and medicine. This fact, in the judgment of the writer, himself a medical man, will probably be the rock on which that Association will finally founder and go to pieces, unless a bridge can be found by which the seceders may return with honour and advantage to the parent association.

It seems to us an absolutely fatal mistake to shut out lay hospital managers from the administration of an organisation having for its object the training and advancement of hospital nurses. Without lay supporters and managers, hospitals themselves could not exist for a single year; how, then, can hospital nurses see it to be their interest to give a slap in the face to those on whom both they and their hospitals so largely depend? The public, it is certain, will never support hospitals unless it can have a voice in their management, any more than the Parliamentary constituencies would pay rates and taxes if they were deprived of their votes. Taxation and representation invariably go together, as do voluntary gifts and control over the use of those gifts. No better illustration of what the lay element is prepared to do for nurses as well as for hospitals can be given than the case of the National Pension Fund. Within a few weeks a number of laymen—friends of hospitals—have not only deposited twenty thousand pounds to make that fund secure, but they, with others, have already subscribed more than six thousand pounds additional on behalf of the Bonus Fund. And yet it is these very laymen that the British Nurses Association rigidly excludes from its councils and its work. This is surely a suicidal policy of the most determined kind. Compared with it, the conduct of the angry dame who killed the goose which laid the golden eggs, was wisdom itself.

The Hospitals Association, as the embodiment of the principles of co-operation and unity amongst all classes of workers in the hospital field, elected a representative committee some months ago to confer with the managers of all the Nurse Training Schools throughout the country, and to ascertain their views and wishes on the subject of registration. This committee has been quietly gathering facts, and has wisely avoided the premature publication of any scheme of registration. Besides, the more prudent of the managers of the Nurse Training Schools see clearly that it is within their power, and may be their wisest course, to register the nurses who receive certificates at their hands, as well as to train and examine them. They recognise that to register their own carefully-selected and highly-trained nurses will save their pupils and themselves from many serious risks which may arise should they give up this privilege to any outside body whatever. In these circumstances the views of the managers of the largest nurse-training schools must first be ascertained and published before any matron or nurse—or, indeed, before the public or anybody—can determine what is the best course of action on this question. At the present moment the wisest policy to pursue is to keep the purse-strings tight and await the guidance of events. Meanwhile we are given to understand that the Report of the Special Committee of *The Hospitals Association* may be looked for shortly, and that it will be issued with the concurrence and approval of several of the largest nurse-training schools in the metropolis.

The scheme of the British Nurses Association is before us. (See page 397 of The Hospital for March 10th.) It is impossible at the close of an article to adequately criticise it in detail, but two of its more prominent features may receive brief comment. First and foremost of these is the exclusion of lay hospital supporters and managers already mentioned. The effect of this, whether intended or not, will be to alienate the sympathy and help of every such manager in Great Britain from nurses as a body and from the nursing staff of each hospital in particular. If nurses, like homœopathic practitioners, voluntarily cut themselves off from the general body of hospital administrators, they inevitably place themselves in a position of antagonism to that body. They in fact assume the place of a trades union, and compel hospital managers to take that of employers, whose interests are separate and distinct. We have nothing whatever to say against trades unions for large masses of operatives, many of whose employers are entirely destitute of sympathy with them and their peculiar difficulties. But we cannot for a moment consider it other than deplorable that sick nurses, who have every hospital manager's warmest sympathy and most practical goodwill, should drift into that position, and with the position should develop, as they inevitably will, the feelings, habits, and objects which always have been, and always must be, characteristic of such unions. The ultimate effect of such a schism, if persisted in, will be to drive a wedge into the organisation of every separate hospital in the kingdom which will split the institution in two.

The next point in the Registration Scheme to which attention should be seriously directed is the constitution of the governing bodies. These will consist of a General Council and an Executive Committee. The area from which the Council is to be drawn is not defined with any precision; but the almost exclusively metropolitan character of the Executive Committee leaves no manner of doubt as to the degree of centralisation which characterises the movement. According to the present programme, London is everything; and the provinces, with Scotland and Ireland, are practically extinguished. London contains in round numbers five millions of inhabitants with hospital accommodation. Ireland contains six millions, and Scotland more than four; yet on the Executive Committee London will have more than twenty matrons (if they join the scheme), whilst Scotland will have but a single one, and Ireland one. When it is remembered that the University of Edinburgh alone has a medical school associated with it larger than any two of the London medical schools put together, and that there are at least five other large medical schools in Scotland, each with its hospital and complete staff of nurses, it will be seen how little likely Scotland is to give its adhesion to any such scheme. The same holds good of Ireland and the provinces. Whilst London, with its population of five millions, is to have more than twenty matrons on the committee, the provinces, with a population of fifteen millions, are to have only five.

The scheme of the British Nurses Association is in truth local, not imperial; parochial, not statesmanlike. In its present form it cannot commend itself either to nurses, doctors, or hospital managers. Still less will it be approved by the intelligent public, who will criticise it entirely on its merits, and quite apart from any other consideration whatsoever. If it be attempted to force it upon Government without such changes as would constitute its complete reconstruction, it will be opposed by the provinces, by Scotland, by Ireland, and, with one consent, by every lay manager in the metropolis. It may be expected that an overwhelming majority of nurses, both in town and country, will refuse altogether to be parties to such a policy of division and self-destruction. Against the combination of nurses into an association for mutual help we have nothing to say. Rather would we urge them by all means so to combine and to form in every hospital a society which shall include, not only nurses and doctors, but every responsible lay manager who shall desire to give his sympathy and practical support. But whilst expressing our entire approval of union and co-operation for mutual advantage and efficiency, we cannot but affirm our conviction that the particular scheme brought forward by the British Nurses Association is based upon principles of isolation and self assertion unworthy of a great movement, and that if it is persisted in as it stands, it will end in disappointment and disaster both to nurses, doctors, patients, and hospitals.

